# Deep generative model of the distal tibial classic metaphyseal lesion in infants: assessment of synthetic images

**DOI:** 10.1093/radadv/umae018

**Published:** 2024-07-04

**Authors:** Shaoju Wu, Sila Kurugol, Paul K Kleinman, Kirsten Ecklund, Michele Walters, Susan A Connolly, Patrick Johnston, Andy Tsai

**Affiliations:** Department of Radiology, Boston Children’s Hospital, Harvard Medical School, Boston, MA 02115, United States; Department of Radiology, Boston Children’s Hospital, Harvard Medical School, Boston, MA 02115, United States; Department of Radiology, Boston Children’s Hospital, Harvard Medical School, Boston, MA 02115, United States; Department of Radiology, Boston Children’s Hospital, Harvard Medical School, Boston, MA 02115, United States; Department of Radiology, Boston Children’s Hospital, Harvard Medical School, Boston, MA 02115, United States; Department of Radiology, Boston Children’s Hospital, Harvard Medical School, Boston, MA 02115, United States; Department of Radiology, Boston Children’s Hospital, Harvard Medical School, Boston, MA 02115, United States; Department of Radiology, Boston Children’s Hospital, Harvard Medical School, Boston, MA 02115, United States

**Keywords:** child abuse, classic metaphyseal lesion, generative models, machine learning, radiography, skeletal survey

## Abstract

**Background:**

The classic metaphyseal lesion (CML) is a distinctive fracture highly specific to infant abuse. To increase the size and diversity of the training CML database for automated deep-learning detection of this fracture, we developed a mask conditional diffusion model (MaC-DM) to generate synthetic images with and without CMLs.

**Purpose:**

To objectively and subjectively assess the synthetic radiographic images with and without CMLs generated by MaC-DM.

**Materials and Methods:**

For retrospective testing, we randomly chose 100 real images (50 normals and 50 with CMLs; 39 infants, male = 22, female = 17; mean age = 4.1 months; SD = 3.1 months) from an existing distal tibia dataset (177 normal, 73 with CMLs), and generated 100 synthetic distal tibia images via MaC-DM (50 normals and 50 with CMLs). These test images were shown to 3 blinded radiologists. In the first session, radiologists determined if the images were normal or had CMLs. In the second session, they determined if the images were real or synthetic. We analyzed the radiologists’ interpretations and employed t-distributed stochastic neighbor embedding technique to analyze the data distribution of the test images.

**Results:**

When presented with the 200 images (100 synthetic, 100 with CMLs), radiologists reliably and accurately diagnosed CMLs (kappa = 0.90, 95% CI = [0.88-0.92]; accuracy = 92%, 95% CI = [89-97]). However, they were inaccurate in differentiating between real and synthetic images (kappa = 0.05, 95% CI = [0.03-0.07]; accuracy = 53%, 95% CI = [49-59]). The t-distributed stochastic neighbor embedding analysis showed substantial differences in the data distribution between normal images and those with CMLs (area under the curve = 0.996, 95% CI = [0.992-1.000], *P *< .01), but minor differences between real and synthetic images (area under the curve = 0.566, 95% CI = [0.486-0.647], *P *= .11).

**Conclusion:**

Radiologists accurately diagnosed images with distal tibial CMLs but were unable to distinguish real from synthetically generated ones, indicating that our generative model could synthesize realistic images. Thus, MaC-DM holds promise as an effective strategy for data augmentation in training machine-learning models for diagnosis of distal tibial CMLs.

**Summary**

The recently developed mask conditional diffusion model generated realistic, diagnostic-quality synthetic radiographic images that radiologists could not differentiate from real images.

**Key Results**

- When presented with 200 radiographs (100 synthetic, 100 with classic metaphyseal lesion [CML]), 3 radiologists were reliable and accurate in differentiating between normal and distal tibial CMLs (kappa = 0.90, accuracy = 92%), but were unreliable and inaccurate in differentiating between real and synthetic images (kappa = 0.05, accuracy = 53%).
- The t-distributed stochastic neighbor embedding analysis showed substantial differences in the data distribution between normal images and those with CMLs (AUC = 0.996, *P* < .01), but minor differences between real and synthetic images (AUC = 0.566, *P* = .11).

## Introduction

Child abuse is a major public health problem. Infants (≤1 year old) are particularly vulnerable and constitute 42.4% of children who die from inflicted injuries.^1^ Most are found to have fractures at the time of death. One highly specific fracture of infant abuse is the classic metaphyseal lesion (CML).^2–5^ This unique fracture extends through the junction of the primary and secondary spongiosa to undercut the subperiosteal bone collar.^6–9^

The CML can pose a diagnostic challenge to radiologists because of its variable and frequently subtle radiographic appearances. This injury can occur in any of the long bones, but the distal tibia is one of the most common sites.^7^^,^^10^^,^^11^ In a prior study, a machine-learning algorithm was used to detect distal tibial CMLs with a sensitivity and specificity of 88% and 96%, respectively.^12^ One of the limitations of this prior study was a cohort of only 73 CML images, which were carefully curated from a 12.5-year experience at a large children’s hospital. One potentially effective strategy to overcome this limitation in model training is to use a data augmentation methodology to increase the size and diversity of the CML database.^13^

Currently, generative adversarial networks (GANs) are the most popular and established deep-learning approach used to generate synthetic images for data augmentation.^14–16^ However, these models can be difficult to train and may generate low-quality and repetitive images when limited data are available.^17^ Recently, a new class of generative model called denoising diffusion probabilistic model has been shown to be effective in generating realistic-appearing synthetic images and has a more straightforward training process.^18^ Furthermore, the denoising diffusion probabilistic model has the ability to incorporate conditional information, including class labels, to generate high-quality conditional images.^18^ We have developed a mask conditional diffusion model (MaC-DM) that incorporates the segmentation masks of the distal tibias and the CML fractures as conditions in generating synthetic images of the distal tibia with and without CMLs.^19^

The next step in moving toward implementation of this approach is to test the verisimilitude of these synthetic images via two approaches: (1) objectively by using a nonlinear dimensionality reduction technique to compare and analyze the feature distribution of the images; and (2) subjectively by asking the expert opinions of radiologists who are readily able to differentiate those distal tibias with CMLs from those without CMLs. Therefore, the aim of this study was to objectively and subjectively evaluate the verisimilitude of the synthetic distal tibial images with and without CMLs that were generated by MaC-DM.

## Methods

Our local institutional review board approved this retrospective study. This study was compliant with the Health Insurance Portability and Accountability Act guidelines, and informed consent was waived.

### Generative model

The methodology we used to generate the synthetic distal tibial radiographs for this investigation is detailed in a prior report.^19^ Briefly, MaC-DM is based on the denoising diffusion probabilistic model^18^ and involves explicitly adding random noise to real images and training a deep learning model to progressively remove this noise. We modified this denoising diffusion probabilistic model by incorporating segmentation masks of the CML fracture region and the distal tibia as conditional information. Our MaC-DM uses these segmentation masks along with a classifier guidance technique^18^ to generate class-specific synthetic radiographs (ie, with or without CML fractures). The detailed architecture of MaC-DM is shown in Figure S1.

### Study material

**Figure 1.**
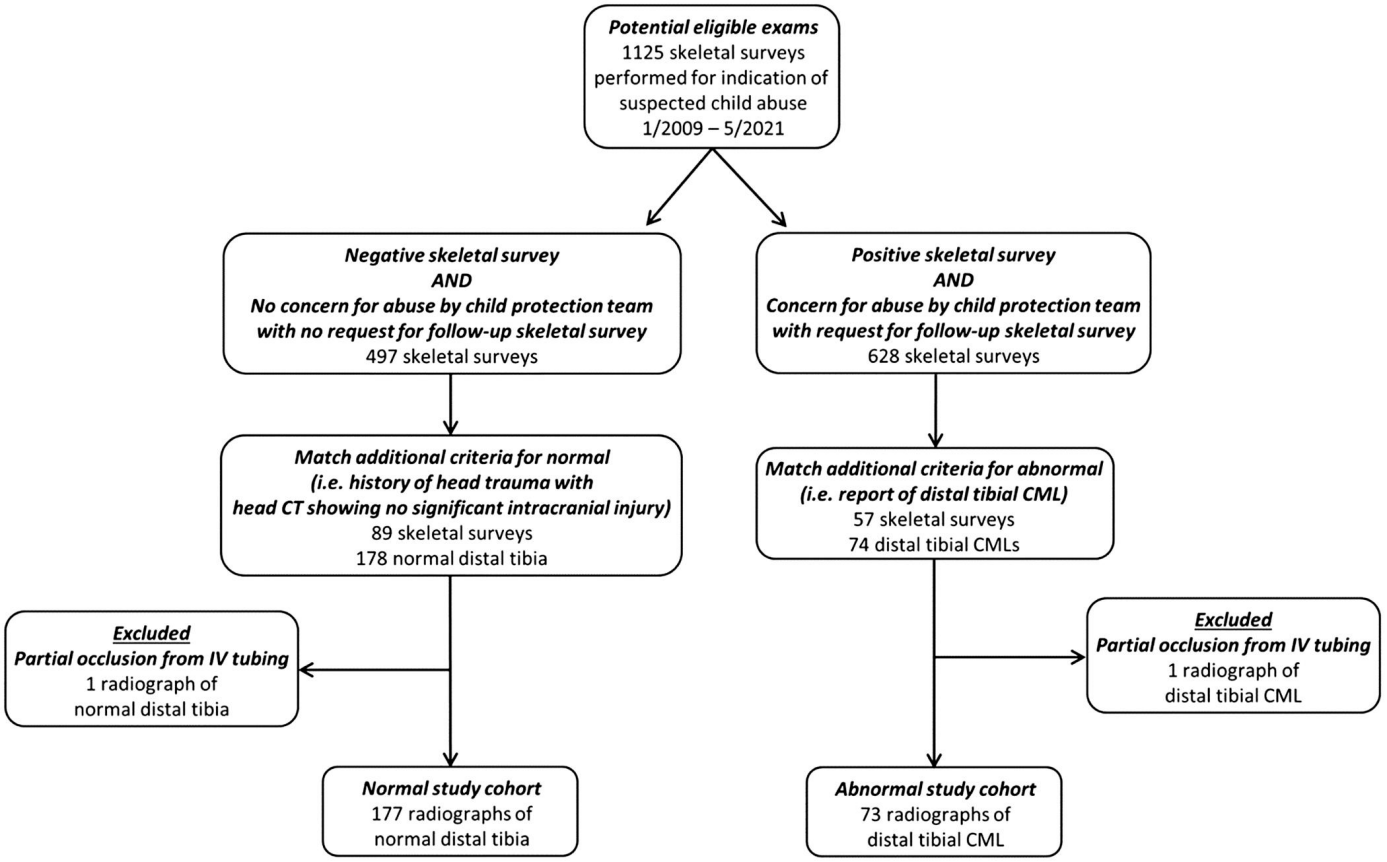
Flow chart illustrating the identification of the normal and the abnormal radiographs from which the testing cohort of 200 images (100 abnormal, 100 synthetic) were derived.

We used a previously assembled database of distal tibial images with and without CMLs^12^ collected over a 12.5-year period (January 2009-May 2021) from a large tertiary children’s hospital. The images were tightly cropped and manually aligned anteroposterior radiographs of the distal tibia from infants with suspected abuse. Specifically, the original 1024 × 1024 grayscale tibial radiographs were cropped to the distal fifth of the tibia using MATLAB 8.3 (MathWorks, Natick, MA), resized to 256 × 256 pixels, and saved in JPEG format by an experienced pediatric radiologist (A.T.). There were 2 different sets of images curated in this database: distal tibial radiographs without CMLs (the normal dataset) and with CMLs (the abnormal dataset). The inclusion criteria for the normal dataset were: (1) negative skeletal survey (excluding simple skull fracture); (2) a clinical history of head trauma that initiated the skeletal survey; (3) a concurrent head CT showing no significant intracranial injury (excluding focal extra-axial blood underneath a skull fracture, if any); (4) no concern for abuse by the child protection team; (5) no follow-up skeletal survey; and (6) no other risk factors for abuse. These infants were considered to be at low risk of abuse. The inclusion criteria for the abnormal dataset were: (1) positive skeletal survey (in addition to a distal tibial CML); (2) a final report indicating a distal tibial CML with subsequent confirmation by the senior author; (3) high-risk of abuse as determined by the child protection team; and (4) a follow-up skeletal survey. Selection of the normal and abnormal study cohorts are shown in Figure 1.

The resultant curated database consisted of 250 distal tibial radiographs. The normal subset of this database consisted of 177 radiographs from 89 infants (male = 61, female = 28; mean age = 5.0 months, SD = 3.3 months, range = 0.2-11.6 months). The abnormal subset of this database consisted of 73 radiographs from 35 infants (male = 24, female = 11; mean age = 3.3 months, SD = 2.9 months, range = 0.4-12 months). From this curated distal tibia image database, we randomly chose 100 real images (50 normal and 50 with CMLs) from 39 infants (male = 22, female = 17; mean age = 4.1 months; SD = 3.1 months) to include in our testing database. Next, for each real normal image in our curated database (N = 177), we used MaC-DM to generate 1 synthetic normal and 1 synthetic CML image based on the input binary class condition (eg, normal, CML). We then randomly selected 50 normal and 50 CML images from this resulting pool of 354 synthetic images (177 real normal images × 2 synthetic images generated per real normal image). These synthetic images were 256 × 256 pixels in size and saved in JPEG format. In the end, our testing database consisted of 200 images (with equal numbers of real normal, real CML, synthetic normal, and synthetic CML images).

### Latent feature distribution of the testing database

To visualize our complex high-dimensional imaging data, we used the t-distributed stochastic neighbor embedding (t-SNE).^20^ This nonlinear dimensionality reduction technique embeds high-dimensional data into a low-dimensional space (2 or 3 dimensions) for optimal data visualization, direct comparison, and statistical analysis of both real and synthetic images. For this technique, we first used the aforementioned real CML dataset (N = 250, with 73 CML images and 177 normal images, all cropped and resized to 256 × 256 during the preprocessing step) to train a ResNet-34 model with 5-fold cross validation—using 80% of the data for training and 20% for testing in each fold.^19^ Then, the trained ResNet-34 model that achieved the best cross-validation performance was selected for feature extraction. We used the second-to-last layer (average pooling layer) of the trained ResNet-34 model to derive the 2-dimensional latent feature representations of the curated real CML dataset. Using this derived latent feature space, we applied the t-SNE method to our testing database (N = 200) and projected the latent features of this database onto a 2-dimensional plane for visualization, comparison, and analysis.

### Image interpretation

Three board-certified radiologists with 30 years (radiologist #1 [R1]), 27 years (radiologist #2 [R2]), and 14 years (radiologist #3 [R3]) of postfellowship experience in pediatric musculoskeletal imaging were blinded to patient demographics, diagnosis, study design, and image acquisition methods. In the first reading session, the radiologists independently determined if the randomly ordered distal tibial images within the testing database were normal or had CMLs. In this first session, they were not informed that the testing image database included synthetic images. In the second session conducted more than a month later, they were informed that the testing image database included synthetic images, and they were asked which of these same, rerandomized images were real and which were synthetic images. The radiologists viewed these images using Windows 10 Microsoft Photos.

### Statistical analyses

#### Evaluation of latent feature space

Receiver operating characteristic (ROC) analysis was used to objectively compare the measured feature distributions in the latent space for (1) normal versus CML images and (2) real versus synthetic images. For each comparison, the area under the curve (AUC) statistic was obtained from the predicted probabilities of a logistic regression model fit by penalized maximum likelihood using SAS’s LOGISTIC procedure (21). The predictors in this model were obtained from the two-dimensional t-SNE features described previously. In this context, AUC measured the probability that the predicted values of one distribution was greater than another (AUC = Prob(pred1 > pred2)). An AUC of 0.5 indicates two perfectly overlapping distributions; an AUC of 1.0 indicates two perfectly distinct distributions, and thus an AUC near 1.0 indicates “difference.”

The AUC analysis was supplemented by the parametric multivariate t-test (SAS’s GLM procedure^21^) and the permutational multivariate analysis of variance (PERMANOVA^22^) nonparametric test to detect differences between the bivariate distributions. We conducted pre- and posttest power calculations to ensure that the 200 images in our testing database was sufficient to detect these differences with reasonable statistical power (details in Supplementary Material S2).

#### Evaluation of radiologists’ interpretations

For the interpretation of the radiologists, we assessed inter-reader agreement via the kappa statistic (scale –1 to 1) using the following guideline^23^: <0.0 (poor agreement), 0.0-0.2 (slight agreement), 0.2-0.4 (fair agreement), 0.4-0.6 (moderate agreement), 0.6-0.8 (substantial agreement), and 0.8-1.0 (almost perfect agreement). To further assess inter-rater reliability, we calculated Krippendorff's Alpha (scale –1 to 1).^24^^,^^25^ For this reliability metric, we considered Alpha >0.8 as having good agreement.^24^

To assess the performances of each of the three radiologists, we constructed 2 × 2 contingency tables for each to assess their ability to distinguish (1) normal versus CML images and (2) real versus synthetic images. To combine results over the 3 radiologists, we estimated sensitivity and specificity using separate population average (marginal) models to accommodate the likely positive within-reader correlation.^26^ These models used an exchangeable variance matrix and were fitted using SAS’s GLIMMIX procedure^21^. Accuracy was derived from these independent sensitivity and specificity estimates (details in Supplementary Material S4). In addition, we used hierarchical summary receiver operating characteristic methods^27^ and utilized the meta-analysis tool^28^ to compute the ensemble estimates of sensitivity and specificity. Hierarchical summary ROC accounts for within-reader correlation when combining results from the three different radiologists.

To assess within-image agreement, we analyzed how consistently and accurately the radiologists were in classifying each image. Specifically, for the two classification tasks (normal versus CML, and real versus synthetic), we collected the three radiologists’ ensemble interpretation of each image, and grouped them into four categories: 0, no radiologist agreed with the ground truth; 1, one radiologist agreed with the ground truth; 2, two radiologists agreed with the ground truth; and 3, three radiologists agreed with the ground truth. We used this methodology as a surrogate to identify the perceived level of difficulty in classifying these images.

## Results

**Figure 2.**
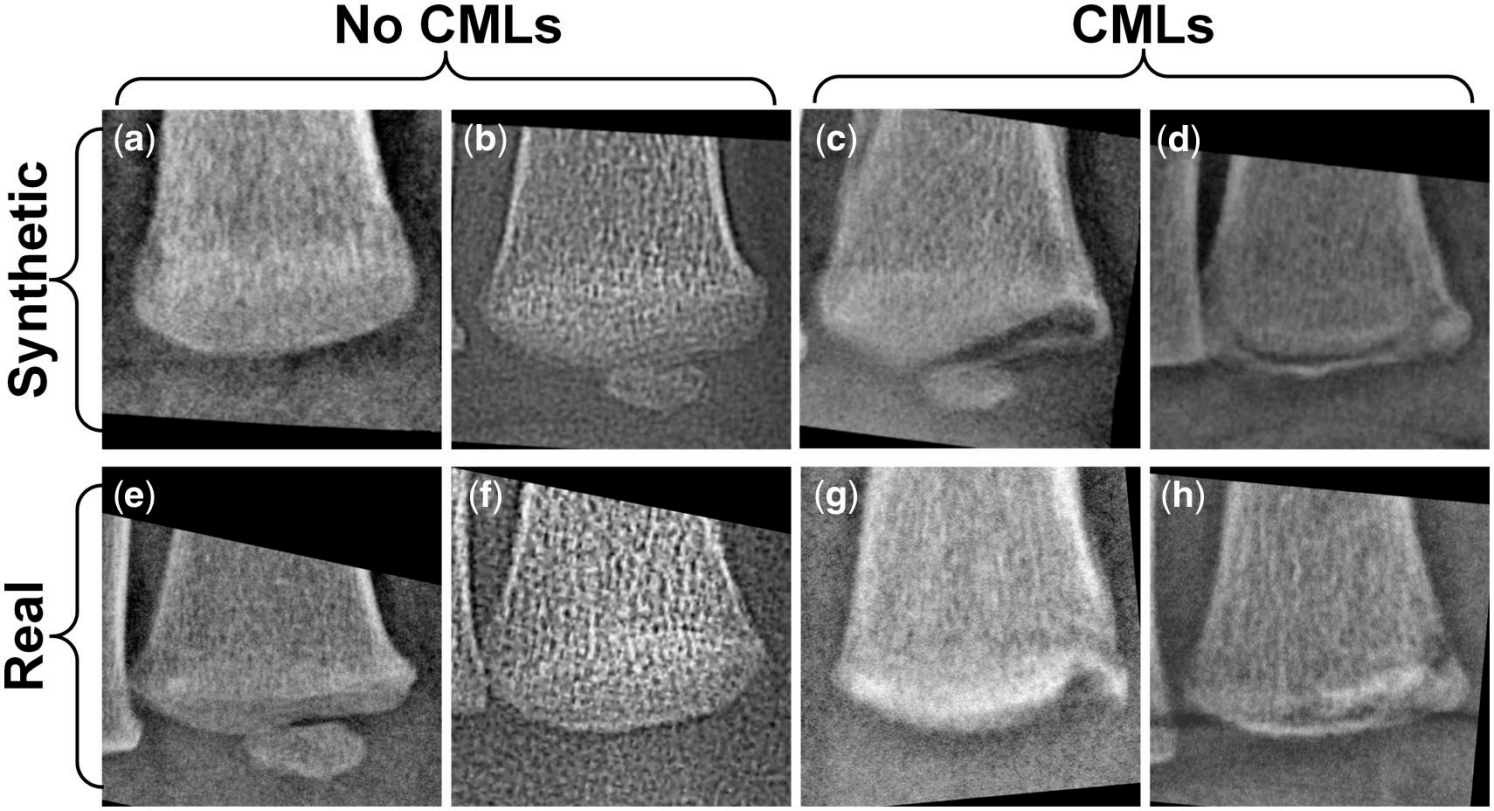
Examples of synthetic (A, B, C, D) and real (E, F, G, H) distal tibial radiographic images, with classic metaphyseal lesions (CMLs) (C, D, G, H) and without CMLs (A, B, E, F). The interpretations of these eight distal tibial images by the three radiologists are presented in Supplemental Material S3.

Examples of real and synthetic distal tibial images with and without CMLs are shown in Figure 2. The three radiologists’ corresponding assessments are shown in Supplementary Material S3.

### Evaluation of latent feature space

**Figure 3.**
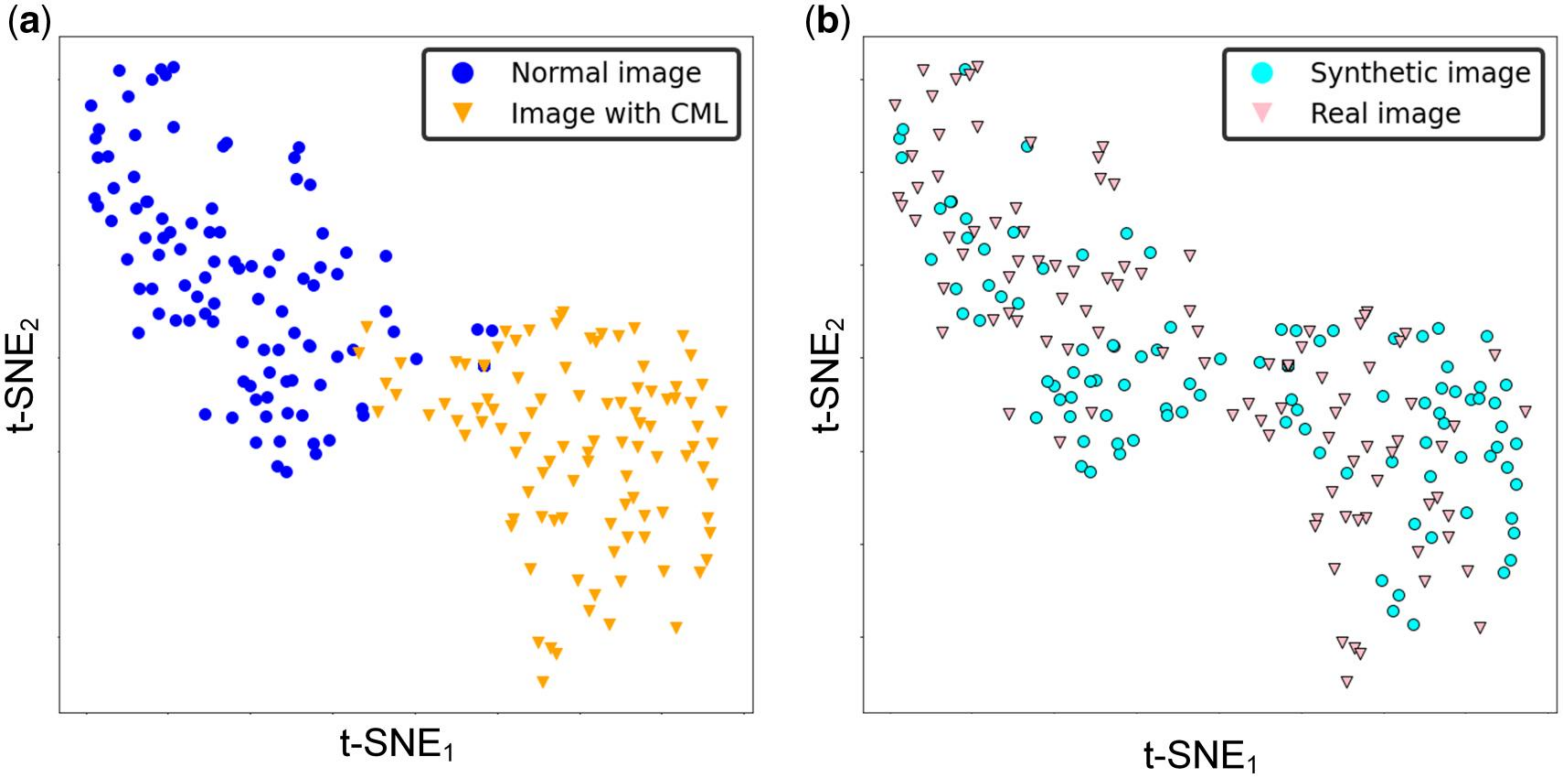
T-distributed stochastic neighbor embedding (t-SNE) of the testing database (N = 200). (A) Two data clusters are shown in the two-dimensional latent feature space, with one cluster corresponding to normal distal tibial images and the other cluster corresponding to the distal tibial images with CMLs. The spatial separation of these two clusters in the latent space (with minimal overlap) suggested discernible differences in the imaging appearances of these two types of images. (B) In contrast, the substantial overlap in the data distributions of the synthetic and real images within the two-dimensional latent feature space indicated mark similarities in the imaging appearances of real and synthetic images.

**Figure 4.**
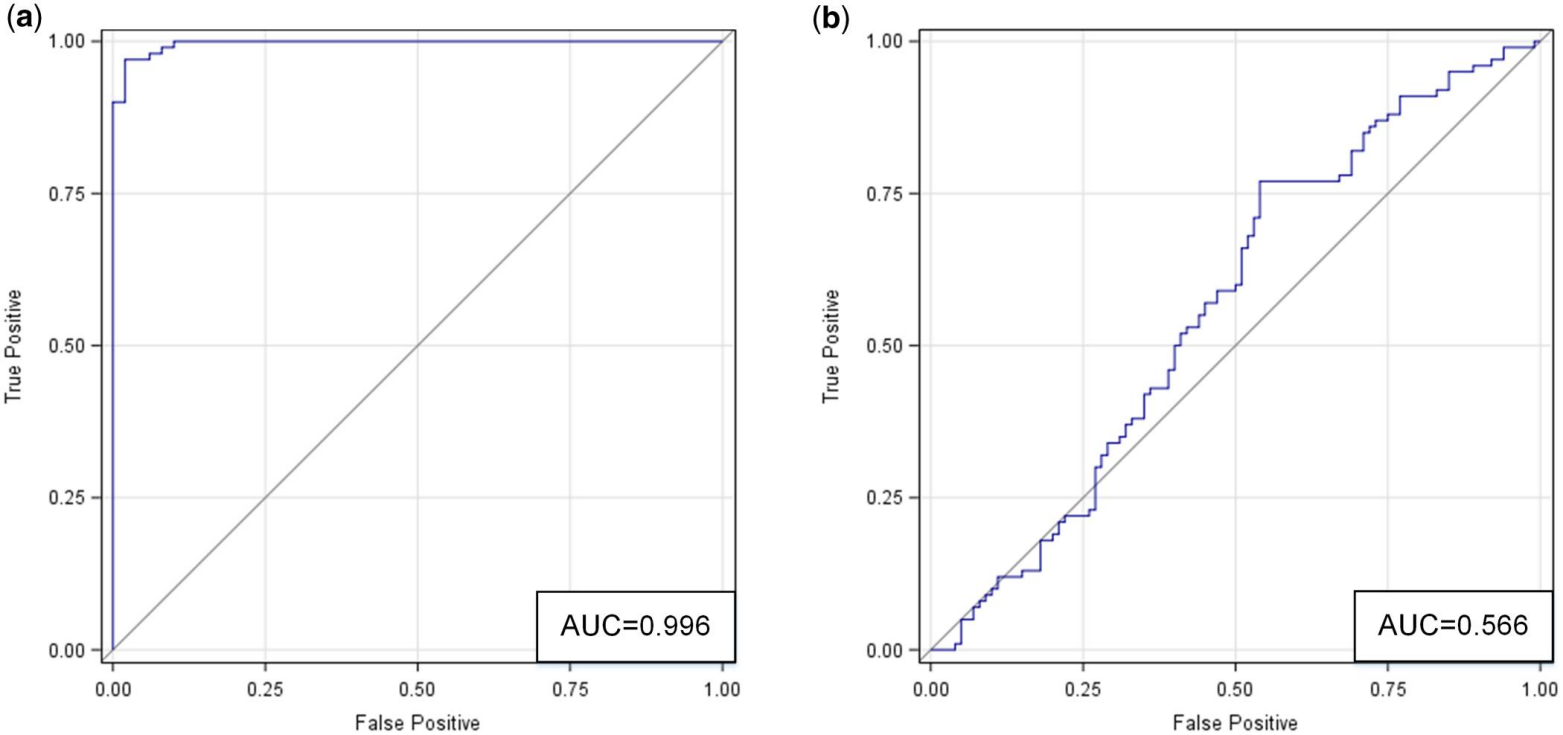
ROC curves for comparing two data distributions in the latent feature space (as derived from t-SNE): (A) classic metaphyseal lesion (CML) versus normal; and (B) real versus synthetic. Abbreviations: ROC = receiver operating characteristic, t-SNE = t-distributed stochastic neighbor embedding.

Using t-SNE, we visually compared the data distributions of normal distal tibial images and those with CMLs, as well as the data distributions of those images that are real and synthetic (Figure 3). For the former, we observed two distinct clusters in the two-dimensional latent feature space, one corresponding to images with CMLs and one corresponding to normal images (Figure 3A). This distinct spatial clustering implied that the data distributions of these two image types were very different and would enable easy partitioning of these two clusters. ROC analysis (Figure 4B) indicated a substantial difference between the two groups (AUC = 0.996, 95% CI = [0.992-1.000]). These results were supported by multivariate tests for group difference (*P *< .001 for both parametric t-test and nonparametric PERMANOVA).

In contrast, there was substantial overlap in the latent feature distributions of real and synthetic images, indicating similarity in their imaging appearances (Figure 3B). And as such, the two image types would be difficult to discriminate. ROC analysis (Figure 4B) showed that the difference between the two distributions was small, close to 0.5 (AUC = 0.566, 95% CI = [0.486-0.647]). Consistent with this finding, multivariate statistical tests for group difference were not statistically significant (parametric t-test [*P *= .21], nonparametric PERMANOVA [*P *= .11]).

### Evaluation of radiologists’ interpretations

Overall, reader agreement in differentiating normal images from those with CMLs were good/excellent (kappa = 0.90, 95% CI = [0.88-0.92]; Krippendorff's Alpha = 0.90, 95% CI = [0.85-0.94]), regardless of whether the images were real (kappa = 0.84, 95% CI = [0.81-0.87]; Krippendorff's Alpha = 0.84, 95% CI = [0.75-0.92]), or synthetic (kappa = 0.96, 95% CI = [0.93-0.99]; Krippendorff's Alpha = 0.96, 95% CI = [0.91-1.00]).

**Figure 5.**
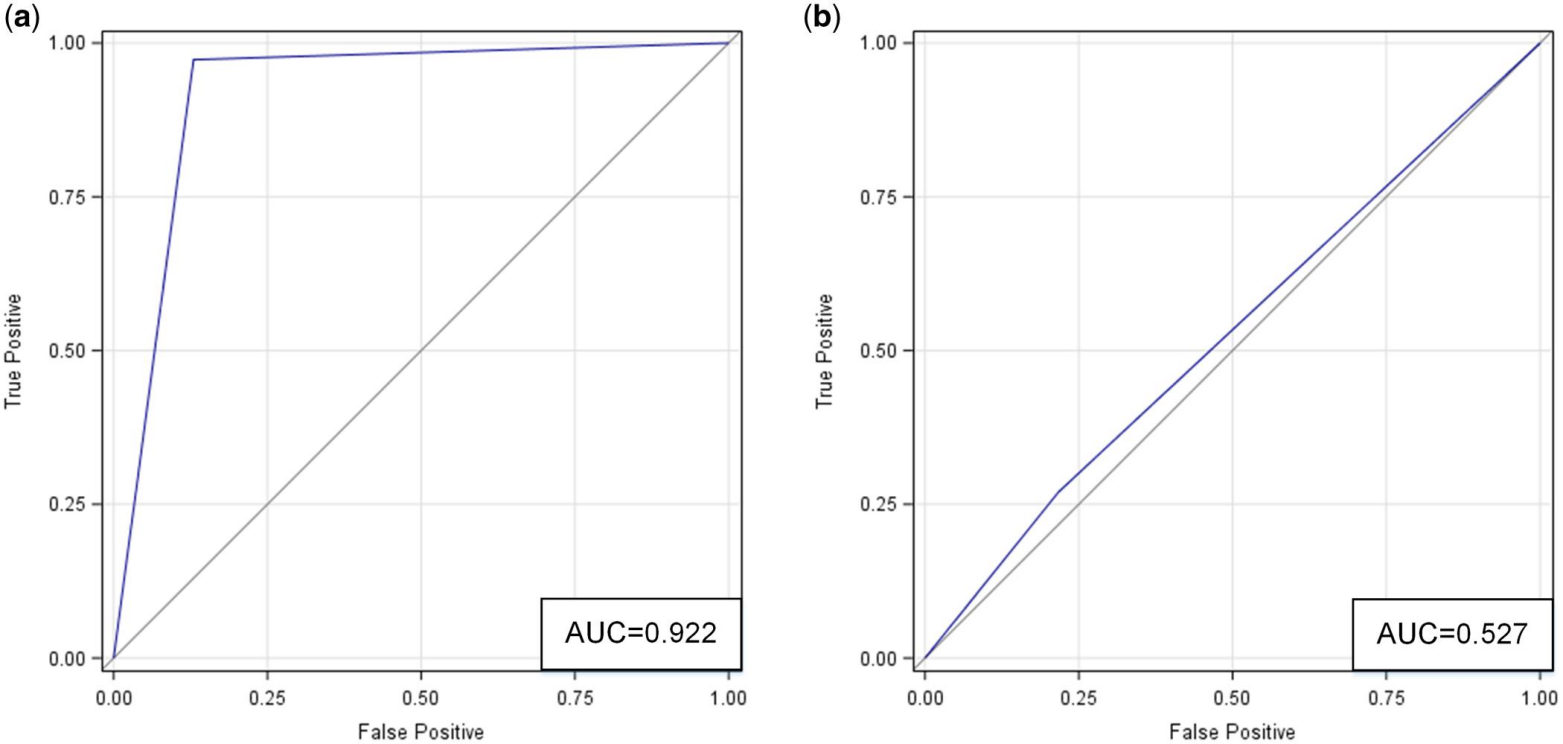
ROC curves based on correlated models that combined data from all three radiologists in the two classification tasks: (A) classic metaphyseal lesion (CML) versus normal; and (B) real versus synthetic. Abbreviation: ROC = receiver operating characteristic.

The ROC analysis (Figure 5A) shows the accuracy (AUC) based on correlated models that combined all readers. Radiologists accurately differentiated normal images from those with CMLs (accuracy = 92%, 95% CI = [89-97]). For the subset of real images, they achieved an accuracy of 90% with 95% CI = [84-95]. For the subset of synthetic images, they achieved an accuracy of 94% with 95% CI = [92-100]. These and additional results are summarized in Tables 1, 3, and 4.

**Table 1.** Performance of the three radiologists in determining whether or not the images have classic metaphyseal lesions (CMLs).

Image with CML?	Radiologist 1		Radiologist 2		Radiologist 3	
**True positive**	All images: 98		All images: 97		All images: 96	
	Real: 48	Synthetic: 50	Real: 47	Synthetic: 50	Real: 46	Synthetic: 50
**True negative**	All images: 89		All images: 86		All images: 84	
	Real: 43	Synthetic: 46	Real: 43	Synthetic: 43	Real: 40	Synthetic: 44
**False positive**	All images: 11		All images: 14		All images: 16	
	Real: 7	Synthetic: 4	Real: 7	Synthetic: 7	Real: 10	Synthetic: 6
**False negative**	All images: 2		All images: 3		All images: 4	
	Real: 2	Synthetic: 0	Real: 3	Synthetic: 0	Real: 4	Synthetic: 0
**Sensitivity**	All images: 0.980		All images: 0.970		All images: 0.960	
	Real: 0.960	Synthetic: 1.000	Real: 0.940	Synthetic: 1.000	Real: 0.920	Synthetic: 1.000
**Specificity**	All images: 0.890		All images: 0.860		All images: 0.840	
	Real: 0.860	Synthetic: 0.920	Real: 0.860	Synthetic: 0.860	Real: 0.800	Synthetic: 0.880
**Accuracy**	All images: 0.935		All images: 0.915		All images: 0.900	
	Real: 0.910	Synthetic: 0.960	Real: 0.900	Synthetic: 0.930	Real: 0.860	Synthetic: 0.940
**F1 score**	All images: 0.938		All images: 0.919		All images: 0.906	
	Real: 0.914	Synthetic: 0.962	Real: 0.904	Synthetic: 0.935	Real: 0.868	Synthetic: 0.943

**Table 2.** Performance of the three radiologists in determining whether the distal tibial radiographic images were real or synthetic.

Real image?	Radiologist 1		Radiologist 2		Radiologist 3	
**True positive**	All images: 2		All images: 45		All images: 34	
	Normal: 0	CML: 2	Normal: 23	CML: 22	Normal: 15	CML: 19
**True negative**	All images: 88		All images: 66		All images: 81	
	Normal: 50	CML: 38	Normal: 39	CML: 27	Normal: 43	CML: 38
**False positive**	All images: 12		All images: 34		All images: 19	
	Normal: 0	CML: 12	Normal: 11	CML: 23	Normal: 7	CML: 12
**False negative**	All images: 98		All images: 55		All images: 66	
	Normal: 50	CML: 48	Normal: 27	CML: 28	Normal: 35	CML: 31
**Sensitivity**	All images: 0.020		All images: 0.450		All images: 0.340	
	Normal: 0.000	CML: 0.040	Normal: 0.460	CML: 0.440	Normal: 0.300	CML: 0.380
**Specificity**	All images: 0.880		All images: 0.660		All images: 0.810	
	Normal: 1.000	CML: 0.76	Normal: 0.780	CML: 0.540	Normal: 0.860	CML: 0.760
**Accuracy**	All images: 0.450		All images: 0.555		All images: 0.575	
	Normal: 0.500	CML: 0.400	Normal: 0.620	CML: 0.490	Normal: 0.580	CML: 0.570
**F1 score**	All images: 0.035		All images: 0.503		All images: 0.444	
	Normal: 0.000	CML: 0.063	Normal: 0.548	CML: 0.463	Normal: 0.417	CML: 0.469

Abbreviation: CML = classic metaphyseal lesion.

**Table 3.** Combined estimates of accuracy, sensitivity, and specificity of three radiologists in the two reading sessions using separate population average (marginal) models.

Mean [95% CI]	First reading session: CML vs. normal			Second reading session: real vs. synthetic		
	Real	Synthetic	Total	CML	Normal	Total
Sensitivity	95% [85%, 98%]	100% [95%, 100%]	97% [92%, 99%]	29% [22%, 37%]	25% [19%, 32%]	27% [22%, 32%]
Specificity	86% [75%, 93%]	88% [77%, 94%]	87% [80%, 92%]	69% [60%, 75%]	88% [81%, 93%]	78% [73%, 83%]
Accuracy	90% [84%, 95%]	94% [92%, 100%]	92% [89%, 97%]	49% [42%, 55%]	57% [54%, 68%]	53% [49%, 59%]

Abbreviation: CML = classic metaphyseal lesion.

**Table 4.** Combined estimates of sensitivity and specificity of three radiologists in the two reading sessions using the hierarchical summary receiver operating characteristic method.

Mean [95% CI]	First reading session: CML vs. normal			Second reading session: real vs. synthetic		
	Real	Synthetic	Total	CML	Normal	Total
Sensitivity	94% [89%, 97%]	99% [95%, 100%]	97% [94%, 98%]	23% [0%, 55%]	11% [11%, 12%]	18% [0%, 57%]
Specificity	84% [77%, 89%]	89% [83%, 93%]	86% [82%, 90%]	69% [56%, 80%]	91% [91%, 92%]	80% [67%, 88%]

Abbreviation: CML = classic metaphyseal lesion.

When the model did not converge because of extreme data (eg, when all readers achieved 100% sensitivity), the dataset was augmented with a single observation (eg, ground truth label = 1, reader predicted label = 0) for each of the three readers.

Overall, reader agreement in differentiating real and synthetic images were poor (kappa = 0.05, 95% CI = [0.03-0.07]; Krippendorff's Alpha = 0.05, 95% CI = [–0.06 to 0.17]). This low level of reader agreement applied equally to images that were normal (kappa = 0.01, 95% CI = [–0.02 to 0.04]; Krippendorff's Alpha = 0.02, 95% CI = [–0.02 to 0.04]) and those with CMLs (kappa = 0.05, 95% CI = [0.02-0.08]; Krippendorff's Alpha = 0.05, 95% CI = [–0.06 to 0.17]).

The ROC analysis (Figure 5B) shows the accuracy (AUC) based on correlated models that combined all readers. Radiologists were inaccurate in differentiating between real and synthetic images (accuracy = 53%, 95% CI = [49-59]. For the subset of normal images, they achieved an accuracy of 57% with 95% CI = [42-55]. For the subset of images with CMLs, they achieved an accuracy of 49% with 95% CI = [49-59]. These and additional results are shown in Tables 2-4.

### Within-image agreement analysis

**Figure 6.**
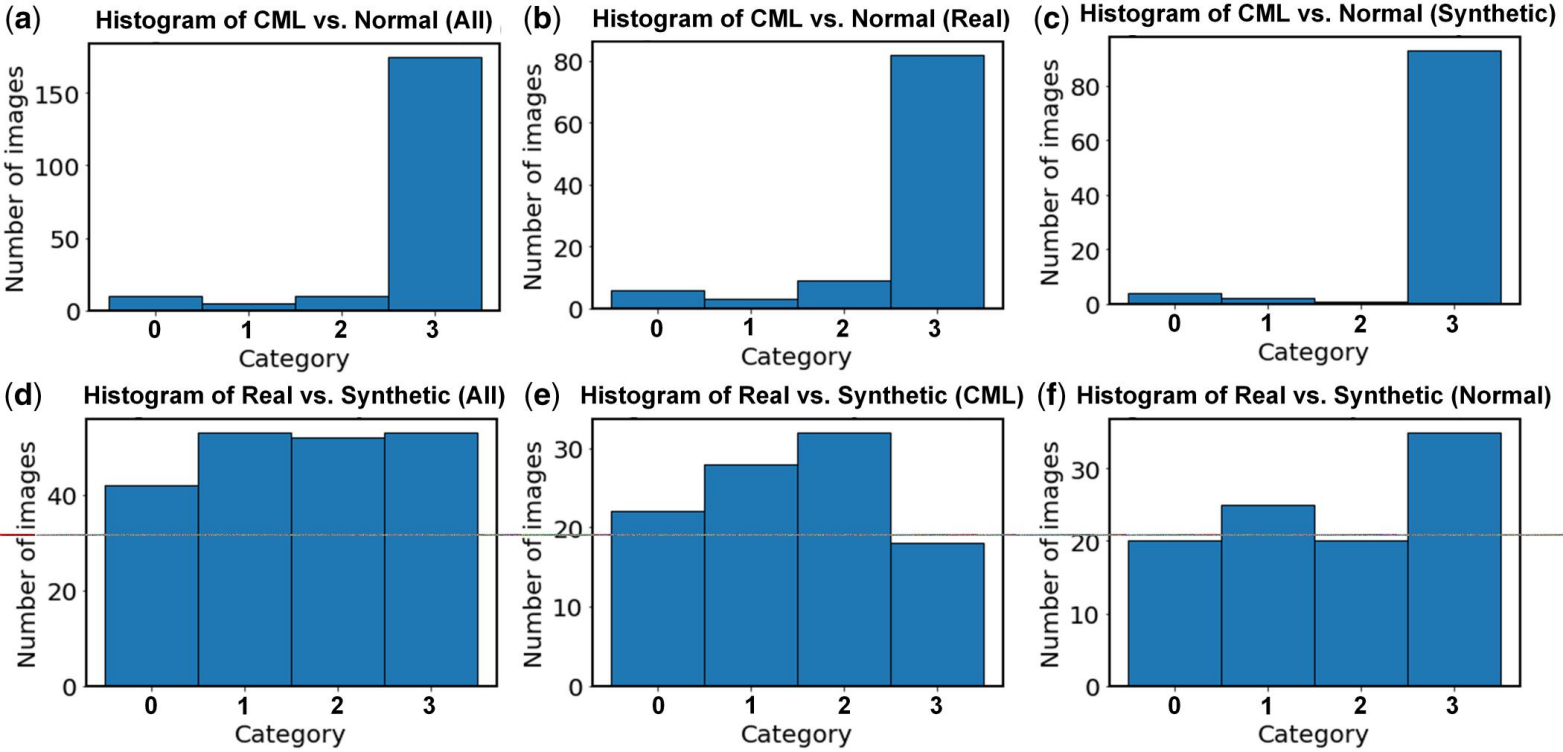
Histogram of the ensemble interpretations by the three radiologists in the two classification tasks: classic metaphyseal lesion (CML) versus normal (top row: A, B, and C), and real versus synthetic (bottom row: D, E, and F). The following four categories were used: 0, no radiologist agreed with the ground truth; 1, one radiologist agreed with the ground truth; 2, two radiologists agreed with the ground truth; 3, three radiologists agreed with the ground truth.

Our analysis revealed a significant difference in accuracy and agreement between the two different classification tasks (Figure 6). In particular, for the radiologists’ ensemble classification of normal versus CML images, >80% of the images were in category 3, indicating that these images were easily classified into normal versus CML by all three radiologists with few disagreements. In contrast, for the radiologists’ ensemble classification of real versus synthetic images, approximately 25% of images were in category 3. In fact, the relatively even distribution across all four categories for this specific task indicated substantial disagreement among radiologists and to the ground truth.

## Discussion

This is, to our knowledge, the first study to evaluate synthetic images created by a generative model in a deep learning-based infant abuse application. Through objective and subjective evaluations of the synthetic images, we found that they were of diagnostic quality and indistinguishable from real images. Specifically, we found that radiologists accurately diagnosed distal tibial images with and without CMLs. This highlights the ability of our diffusion models to generate synthetic images with class-specific features (ie, with or without CML fractures). In contrast, we found that all three radiologists could not reliably determine whether the distal tibial images were real or synthetic, achieving an accuracy ranging from 45% to 58%. This poor performance supports the visual realism achieved by our synthetic images. This overall performance of the radiologists is concordant with the results of the t-SNE analysis that showed that the data distributions of images with and without CMLs were very different, whereas the data distributions of real and synthetic images were quite similar.

Few prior research studies used radiologists to evaluate augmented images produced by generative models, including models based on GANs^29^ and diffusion models.^30^ Prezja et al. proposed GAN-based models to generate synthetic X-ray images of knee joints to aid in classifying the severity of osteoarthritis.^29^ They found that radiologists could not accurately distinguish between real and synthetic images. More recently, Ali et al. used stable diffusion models trained with 3156 X-ray images to generate synthetic lung X-ray images.^30^ They noted that radiologists could accurately distinguish real from synthetic images but that recognizable artifacts on their synthetic images might have aided the radiologist. In contrast, our MaC-DM generated high-quality synthetic images with few and subtle artifacts that are indistinguishable from real images.

An important factor contributing to our success was the integration of segmentation masks within our conditional diffusion model, incorporating regional morphology of the CML fracture and the distal tibia. This strategic integration mitigates the necessity of an extensive training dataset that is generally assumed to be necessary for diffusion models to generate realistic images.

Our approach, which leverages MaC-DM’s capabilities to generate synthetic medical images, has important implications for the field of health care. First, the use of synthetically generated medical images may reduce the reliance on large volumes of real patient data for research and development. For instance, recent studies have found that high-quality synthetic data could be used to improve surgical outcomes in robot-assisted surgery,^31^ to optimize patient-specific treatment plans,^32^ and to improve health care models for disease detection.^33^ For our infant abuse application, synthetic data can be used to increase the size and diversity of our CML database to better train our deep learning-based models for CML detection. Second, realistic synthetic medical images provide a valuable resource for training medical professionals. In particular, by being able to synthetically generate CML fractures with variable patterns (eg, focal vs diffuse or acute versus healing), these synthetic images offer a versatile platform for teaching health care professionals, in detecting CMLs, and in gaining valuable experience with a wide range of CML appearances. Third, synthetically generated medical images can mitigate patient privacy concerns, a particularly important factor in child abuse cases where informed approaches to medico-legal issues and patient confidentiality are vital. Health care providers and researchers can share data for collaborative, teaching, and learning purposes without compromising patient privacy.^34^

Our results should be considered in light of the following limitations. First, we lack a dataset of real images gathered from other medical facilities for external validation. Second, the images used to train MaC-DM require a preprocessing step that included manual orientation and cropping of skeletal survey radiographs by an experienced radiologist—a subjective and time-consuming task. In the future, we intend to use an object detection method, such as YOLO,^35^ to improve the efficiently in collecting real images. Third, although our multivariate tests showed no statistically significant difference between real and synthetic images in the t-SNE space (parametric t-test [*P *= .21], nonparametric PERMANOVA [*P *= .11]), some quantifiable differences may be present. These might arise from the subtle variations in distal tibia structures or background textures. The multivariate tests suggest no significant gap, but minor inherent differences between real and synthetic images likely exist. Fourth, our study used binary response data (real/synthetic) for radiologists’ opinions. A Likert grading scheme (eg, 1-3 synthetic, 4-6 unsure, 7-9 real) could capture larger degrees of uncertainty in the radiologists' opinions. Fifth, our study focused on the distal tibia and further research is required to determine if our approach is applicable to other anatomic regions where CMLs can occur. Finally, our study included only 200 test images with three experienced pediatric radiologists. A future study using a larger number of test images from multiple institutions and readers with more varied training and experiences would be needed to demonstrate the generalizability to our findings.

## Conclusion

Based on objective and subjective evaluations, we found that the MaC-DM generated synthetic distal tibial images, with and without CMLs, that were realistic in appearance and of diagnostic quality. These results suggest that our MaC-DM is a promising strategy for data augmentation in training deep learning models for detection of distal tibial CMLs.

## Supplementary Material

umae018_Supplementary_Data

## Data Availability

The data that support the findings of this study are not openly available because of the sensitive nature of these images, but they are available from the corresponding author on reasonable request. **Code availability**: The computer code associated with this study at: https://github.com/wushaoju/Assessment-of-Synthetic-Images-CMLs.

## Abbreviations

AUC: area under the curve
CML: classic metaphyseal lesion
GAN: generative adversarial network
MaC-DM: mask conditional diffusion model
PERMANOVA: permutational multivariate analysis of variance
ROC: receiver operating characteristic
t-SNE: t-distributed stochastic neighbor embedding
