# Supplementary material for: Deep generative model of the distal tibial classic metaphyseal lesion in infants: assessment of synthetic images
Source: Radiol Adv. 2024 Jul 4;1(2):umae018. doi: 10.1093/radadv/umae018 (PMC11335364; doi:10.1093/radadv/umae018)
Supplement: umae018_Supplementary_Data [file umae018_Supplementary_Data.docx]

**Deep Generative Model of the Distal Tibial Classic Metaphyseal Lesion in Infants: Assessment of Synthetic Images**

**Author:** Shaoju Wu, Sila Kurugol, Paul K. Kleinman, Kirsten Ecklund, Michele Walters, Susan A. Connolly, Patrick Johnston, Andy Tsai

**Address:**

Department of Radiology,

Boston Children’s Hospital,

Harvard Medical School,

300 Longwood Ave, Boston, MA 02115

United States of America

Shaoju Wu (🖂)

Department of Radiology

Boston Children’s Hospital

Harvard Medical School

300 Longwood Avenue

Boston, MA 02115

United States of America

Email: [shaoju.wu@childrens.harvard.edu](mailto:shaoju.wu@childrens.harvard.edu)

**Supplemental Material**

**S1. Flow diagram of masked conditional diffusion model (MaC-DM)**


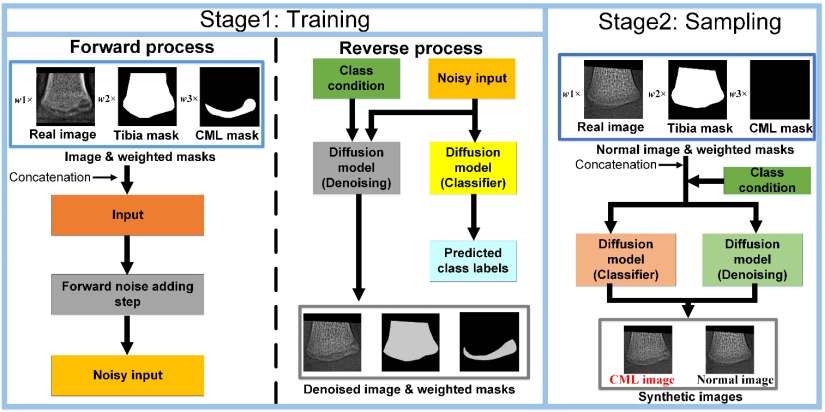


**Figure S1.** Flow diagram of MaC-DM. The training process (Stage1) contains the forward and the reverse process. The forward process involves iteratively introducing noise to both the input images and their corresponding segmentation masks. In contrast, the reverse process involves training two diffusion models: one for image denoising and another for classification. For Stage 2, synthetic images are generated during the sampling process by leveraging the diffusion model, which is guided by the classifier. In order to achieve a balanced intensity range for the three inputs—the real image, the tibia mask, and the CML mask—we employ three weighting factors: *w*_1_, *w*_2_, and *w*_3_.

**S2. Sample size calculation**

For pre-test power analysis, we determined the sample size based on whether the addition of synthetic images to real ones could enhance CML detection. In particular, we made the assumption that if synthetic images were within the data distribution of real ones, they should not decrease and can only improve the performance of CML detection when using the current machine-learning models. Our hypothesis assumed a 12% improvement in sensitivity and specificity when using a ResNet-34 model trained on both real and synthetic images in comparison to a ResNet-34 model trained solely with real images. We estimated the sample size necessary to make these improvements using a two-sided McNemar test for paired proportions, with 95% confidence intervals (CIs) and 80% power. We found that we needed at least 170 images to detect a statistically significant difference, which prompted us to choose a testing database consisting of 200 images.

To ensure the adequacy of the assumptions made in the pre-test power analysis, we conducted a post-test power analysis. Specifically, we compared the CML diagnostic performance of two ResNet-34 classifiers: one trained with only real images (N=100), and another trained with both real and synthetic images (N=200). Both classifiers were evaluated on an unseen distal tibial CML dataset (N=53, with 45 normal and 8 CML images) that were curated using the same inclusion criteria as described in the Methods section but over a different time period (2006-2008 and 2021-2022). This analysis assessed whether the model trained using real and synthetic images generated by our MaC-DM achieved non-inferiority in CML diagnostic performance when compared to the model trained solely on real images. Through this post-test analysis, we found that the ResNet-34 classification model trained with both real and synthetic images achieved a sensitivity of 75.0% on the unseen distal tibial dataset, reflecting a 12.5% improvement compared to the model trained with only real images (which had a sensitivity of 62.5%). This change in sensitivity is in line with the 12.0% improvement assumed in our pre-test power analysis, suggesting that our pre-test assumptions were reasonable.

**S3. Specific interpretations of the radiologists for the selected images**

The interpretations of the three radiologists (R1, R2, and R3) for the 8 representative images shown in **Fig. 2** are listed below:

(a) R1=real normal, R2=synthetic normal, and R3=real normal;

(b) R1=synthetic normal, R2=synthetic normal, and R3=synthetic normal;

(c) R1=real CML, R2=real CML, and R3=synthetic CML;

(d) R1=synthetic CML, R2=synthetic CML, and R3=synthetic CML;

(e) R1=synthetic normal, R2=real normal, and R3=real normal;

(f) R1=synthetic normal, R2=synthetic normal, and R3=synthetic normal;

(g) R1=synthetic CML, R2=real CML, and R3=real CML;

(h) R1=synthetic CML, R2=synthetic CML, and R3=synthetic CML.

**S4. Definition of accuracy, sensitivity, and specificity**

For objective latent space evaluation, AUC was calculated as the area under the empirical ROC curve based on a logistic regression model. The ROC curve itself was the locus of all distinct (sensitivity, 1-specificity) points based on the predicted probabilities for the two groups.

For binary evaluation of the radiologists’ interpretations for a given reader, the 2×2 frequency table of ground truth (0 or 1) versus reader assessment (0 or 1) provides true positive (TP), true negative (TN), false negative (FN), and false positive (FP) numbers (with CML=1 and normal=0 for the CML versus normal reading section; and real=1 and synthetic=0 for the real versus synthetic reading section). Sensitivity, specificity, accuracy, and F1 score were calculated as follows:

- Sensitivity = TP / (TP + FN)
- Specificity = TN / (TN + FP)
- Accuracy = AUC = (specificity + sensitivity)/2.
- F1 score = 2TP / (2TP + FP + FN)

For binary evaluation of the radiologists’ interpretations averaged over the three radiologists, a pair of correlated (population average) logistic models were used to estimate sensitivity and specificity, respectively, one pair for CML versus normal, and another pair for real versus synthetic. To illustrate using the CML versus normal case, sensitivity was estimated as the probability that the assessment was CML using the subset of observations that actually were CML. Similarly, specificity was estimated as the probability that the assessment was normal using the subset of observations that actually were normal. Accuracy was calculated as (specificity + sensitivity)/2, which is the value of AUC under the ROC curve based on the single point (1-sensitivity, specificity) shown in **Fig. 5a**. The 95% confidence interval for accuracy was derived from the confidence interval for the average logodds via the logistic transformation. The (Wald-based) confidence interval for the average logodds itself was based on the variance of the average of two independent logodds estimates.

**S5. Explanation of abbreviations**

| **Abbreviations** | **Meaning** |
| --- | --- |
| AUC | Area under the curve |
| CPT | Child protection team |
| CML | Classic metaphyseal lesion |
| CI | Confidence interval |
| MaC-DM | Mask conditional diffusion model |
| GAN | Generative adversarial network |
| SD | Standard deviation |
| PERMANOVA | Permutational multivariate analysis of variance |
| HSROC | Hierarchical summary receiver operating characteristic |
